# Draft Genome Sequencing of Stenotrophomonas indicatrix BOVIS40 and Stenotrophomonas maltophilia JVB5, Two Strains with Identifiable Genes Involved in Plant Growth Promotion

**DOI:** 10.1128/MRA.00482-21

**Published:** 2021-07-15

**Authors:** Olubukola Oluranti Babalola, Bartholomew Saanu Adeleke, Ayansina Segun Ayangbenro

**Affiliations:** a Food Security and Safety Niche Area, Faculty of Natural and Agricultural Sciences, North-West University, Mmabatho, South Africa; University of Arizona

## Abstract

Here, plant growth-promoting *Stenotrophomonas* strains isolated from the sunflower root endosphere were studied. Bacterial DNA was sequenced on Illumina’s NextSeq platform. The gene prediction reveals diverse functional genes involved in plant growth promotion from each bacterial genome. The exploration of bacterial resources as bioinoculants is promising for agricultural biotechnology.

## ANNOUNCEMENT

*Stenotrophomonas* species are free-living, Gram-negative nonsporeformers that are commonly found in the soil and plant environments (1). *Stenotrophomonas* species can be involved in plant growth promotion (PGP) (2), although some are yet to be explored. Therefore, genomic elucidation can help predict the diverse genes responsible for bacterial functions in agricultural biotechnology.

Bacteria isolated from sunflower roots were sourced from farmlands in Lichtenburg, South Africa (26°4′31.266″S, 25°58′44.442″E), in February 2020. The healthy sunflower plants were carefully uprooted, placed inside sterile ziplock bags, transported to the laboratory, and stored at 4°C. The root samples were cut into small pieces with a sterile scalpel and washed in sterile distilled water. To ensure complete removal of the epiphytic bacteria, surface sterilization was achieved by soaking the samples in 70% ethanol for 3 min, followed by 3% sodium hypochlorite for 3 min, 70% ethanol for 30 s, and rinsing with sterile distilled water. The level of sterility of the samples was assessed by pour plating onto Luria-Bertani (LB) medium using the last water used to rinse the plant samples. One gram of plant material was weighed, suspended in 1 M phosphate-buffered solution, and manually macerated in a mortar and pestle until a smooth suspension was obtained. Sample suspensions were serially diluted up to 10^−9^ dilutions, and 0.1 ml of an aliquot from dilutions 10^−5^ and 10^−6^ was pipetted into petri dishes and pour plated with sterilized LB agar. The inoculated petri plates were incubated at 28°C for 24 h. Distinct bacterial colonies that formed on the plates were counted and selected based on their morphological appearance. A pure culture of the bacterial isolate was obtained by repeated streaking onto sterile LB agar and incubated at 28°C for 24 h. The pure bacterial strains were kept on an agar slant and stored at 4°C for further use.

The pure strains were used for DNA extraction, using a commercial Quick-DNA miniprep kit specific for fungi or bacteria (Zymo Research, Irvine, CA, USA; catalog number D6005). Whole-genome sequencing was performed on Illumina’s NextSeq platform at Inqaba Biotechnical Industries (Pty.) Ltd. (Pretoria, South Africa). The sample preparation (DNA library) was performed using a NextSeq midoutput kit, and a data set (2 × 150-bp paired-end reads) was generated for each sample. Genomic sequences were analyzed on the KBase platform (https://kbase.us/) (3). The quality of the sequence reads was evaluated using FastQC version 0.11.5 (4), while sequence adaptors and low-quality bases were removed using Trimmomatic version 0.36 (5). Furthermore, the sequence reads were assembled using SPAdes version 3.13.0 (6). Gene annotation and prediction were performed using the RASTtk (Rapid Annotations using Subsystems Technology) toolkit version 1.073 and the publicly available NCBI (https://www.ncbi.nlm.nih.gov/) Prokaryotic Genome Annotation Pipeline (PGAP) (7). All analyses were performed using default parameters unless otherwise specified.

**FIG 1:**
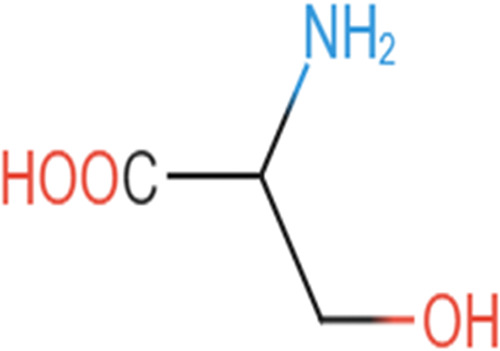
Predicted nonribosomal peptide/polyketide monomers from *Stenotrophomonas* strains BOVIS40 and JVB5.

Secondary metabolites were determined using antiSMASH version 6.0.0 (8). The sequence analysis of strain BOVIS40 yielded a sequence read count of 7,301,524 bp, a genome size of 4,427,090 bp, a G+C content of 66.4%, 4,446 coding sequence genes, 62 tRNAs, and 2 rRNAs. In addition, a sequence read count of 8,764,890 bp, a genome size of 4,771,305 bp, a G+C content of 66%, 57 tRNAs, and 4,160 coding genes were obtained from the genome assembly of strain JVB5. The predicted PGP genes and nonribosomal peptide/polyketide (NRPS/PKS) monomers are presented in Table 1 and Fig. 1, respectively. The detection of PGP traits and secondary metabolites (NRPS/PKS) in strains BOVIS40 and JVB5 will help in understanding the mechanisms employed by bacterial endophytes within the endosphere for plant growth through phytohormone production and the secretion of biocontrol compounds. In addition, the expression of PGP genes in the genomes may modulate their multiple functions for enhancing plant growth for improved crop production. Furthermore, the antibiosis potential of endophytic bacteria against phytopathogens has been linked to their ability to produce NRPS/PKS antimicrobial compounds (9). Hence, the potential function of NRPS/PKS reveals novel information about endophytic bacteria as excellent candidates in ensuring sustainable plant health.

**TABLE 1:** Genome annotation information of plant growth-promoting strains BOVIS40 and JVB5

Trait	Gene	Locus tag_BOVIS40_	Locus tag_JVB5_	Product	Data for strain^*b*^	
					BOVIS40	JVB5
Nitrogen fixation	*ntrC*	J0657_03480	ND^*a*^	Nitrogen regulation protein NR(I)	+	−
	*nifS*	J0657_16100	J0661_17475	Cysteine desulfurase	+	+
	*nifF*	J0657_01315	J0661_20290	Flavodoxin	+	+
Phosphate solubilization	*ppx*	J0657_02585	ND	Exopolyphosphatase	+	−
	*phoU*	J0657_09385	J0661_14530	Phosphate signaling complex protein PhoU	+	+
	*phoA*	J0657_04540	ND	Alkaline phosphatase	+	−
Phosphate transport	*pstS*	J0657_08775 J0657_08780	J0661_14510 J0661_17540	Phosphate ABC transporter substrate-binding protein PstS	+	+
	*pstB*	J0657_09390	J0661_14525	Phosphate ABC transporter ATP-binding protein PstB	+	+
	*pstA*	J0657_09395	J0661_14520	Phosphate ABC transporter permease PstA	+	+
	*pstC*	J0657_09400	J0661_14515	Phosphate ABC transporter permease subunit PstC	+	+
	*agp*	ND	J0661_11895	Bifunctional glucose-1-phosphatase/inositol phosphatase	−	+
Phosphonate degradation	*phnD*	ND	J0661_03670	Phosphonate ABC transporter substrate-binding protein	−	+
Siderophore production	*fiu*	J0657_11665	ND	Catecholate siderophore receptor Fiu	+	−
Tryptophan and IAA production	*trpA*	J0657_13275	J0661_15745	Tryptophan synthase subunit alpha	+	+
	*trpB*	J0657_13265	J0661_15735	Tryptophan synthase subunit beta	+	+
	*trpC*	J0657_01680	J0661_17725	Indole-3-glycerol phosphate synthase	+	+
	*trpD*	J0657_01675	J0661_17720	Anthranilate phosphoribosyltransferase	+	+
	*aldH*	J0657_14110	ND	Aldehyde dehydrogenase	+	–
Cytokinin	*miaA*	J0657_16145	J0661_15225	Adenosine N6-dimethylallyltransferase MiaA	+	+
	*miaB*	J0657_07115	J0661_08550	N6-isopentenyl adenosine-methylthiotransferase MiaB	+	+
Biofilm production	*bcsF*	J0657_13495	ND	Cellulose biosynthesis (CP) protein BcsF	+	−
	*bcsG*	J0657_13500	ND	Cellulose biosynthesis protein BcsG	+	−
	*yhjQ*	J0657_13510	ND	Cellulose synthase operon protein YhjQ	+	−
	*bcsA*	J0657_13515	ND	UDP-forming cellulose synthase catalytic subunit	+	−
	*bcsB*	J0657_13520	ND	CP cyclic di-GMP-binding regulatory protein BcsB	+	−
	*bcsC*	J0657_13525	ND	Cellulose biosynthesis protein BcsC	+	−
	*bcsZ*	J0657_13530	ND	Cellulose	+	−

a ND, not detected.

b +, present; –, absent.

### Data availability.

The draft genome sequences for strains BOVIS40 and JVB5 are available in GenBank under the BioProject accession numbers PRJNA706595 and PRJNA706608, respectively. The Sequence Read Archive (SRA) accession number for strain BOVIS40 is SRR13883846, while that for strain JVB5 is SRR13908543. Genome accession numbers JAGENA000000000 and JAGEKL000000000 were assigned to strains BOVIS40 and JVB5, respectively.
